# Diagnostic and surgical management of primary central nervous system angioleiomyoma: A case report and literature review

**DOI:** 10.3389/fonc.2022.1072270

**Published:** 2022-12-16

**Authors:** Emanuele Rubiu, Emanuele La Corte, Giulio Bonomo, Francesco Restelli, Jacopo Falco, Elio Mazzapicchi, Morgan Broggi, Marco Paolo Schiariti, Bianca Pollo, Valentina Pinzi, Maria Grazia Bruzzone, Francesco Di Meco, Francesco Acerbi, Paolo Ferroli

**Affiliations:** ^1^ Department of Neurosurgery, Fondazione IRCCS Istituto Neurologico Carlo Besta, Milan, Italy; ^2^ University of Milan, Milan, Italy; ^3^ Alma Mater Studiorum University of Bologna, Bologna, Italy; ^4^ Neuropathology Unit, Fondazione IRCCS Istituto Neurologico Carlo Besta, Milan, Italy; ^5^ Radiotherapy Unit, Department of Radiosurgery, Fondazione IRCCS Istituto Neurologico Carlo Besta, Milan, Italy; ^6^ Neuroradiology Department, Fondazione IRCCS Istituto Neurologico Carlo Besta, Milan, Italy; ^7^ Department of Oncology and Hematology-Oncology, University of Milan, Milan, Italy; ^8^ Department of Neurological Surgery, Johns Hopkins Medical School, Baltimore, MD, United States

**Keywords:** angiography, angioleiomyoma, CNS, fluorescein, intracranial, intraoperative confocal endomicroscopy, primitive

## Abstract

Angioleiomyoma (ALM) is a benign smooth muscle neoplasm that mainly occurs in lower extremities subcutaneous tissue and generally affects middle-aged adults. This tumor histotype may rarely localize intracranially, although only a few cases have been described in the literature. We report a case of intracranial ALM, whose differential diagnosis has been particularly challenging, and firstly provide a comprehensive radiological and intra-operative evaluation of a such rare entity. This represents also the first report of the use of intraoperative confocal microscopy in ALM and the first documented short-term recurrence. At this regard, a scoping literature review has been conducted with the aim of presenting the major clinical and diagnostic features along with the proposed therapeutic strategies.

## Introduction

Angioleiomyoma (ALM), also called angiomyoma or vascular leiomyoma, is defined as a benign and indolent soft tissue neoplasm arising from smooth muscle cells. According to the 2016 central nervous system (CNS) tumors classification by World Health Organization (WHO), ALM is classified as a mesenchymal non-meningothelial brain tumor (1). On the contrary, in 2021 WHO classification of CNS tumors, the term “angioleiomyoma” is not mentioned because leiomyoma is now described in the soft tissue tumors category. Microscopically, the disease can be recognized by its pattern of intersecting fascicles, composed of eosinophilic spindle cells with blunt-ended nuclei (2). Considering the lack of mitotic activity and cytological atypia, ALM represents a benign neoplastic entity (2). Diffuse leptomeningeal leiomyoma and an angioleiomyomatous type represent disease variants and have been described in the literature (3). Epstein-Barr Virus (EBV) and AIDS-associated ALMs have also been reported (4). ALM is commonly located in the lower extremities and affects middle-aged adults and usually manifests as an isolated, painful, and solid mass (5). Although ALM may originate from the subcutaneous tissue of the trunk (6), visceral and mucosal locations have been reported (7). On the contrary, primitive intracranial ALM represents an exceedingly rare tumor. Since the first reported case by Lach et al. in 1994, 57 cases have been reported in the literature (8). From these reports, it emerged that primitive CNS ALM is mostly observed in women, with an age peak around the fourth decade (9). Both the imaging features and the intraoperative surgical considerations have been analyzed in the present paper, in which we present the case of a middle-aged woman with a history of fatigue and weakness in the right limbs. Brain magnetic resonance imaging (MRI) revealed the presence of a lesion located in the free left edge of the tentorium, which posed an indication for surgical resection.

## Case report

**Figure 1:**
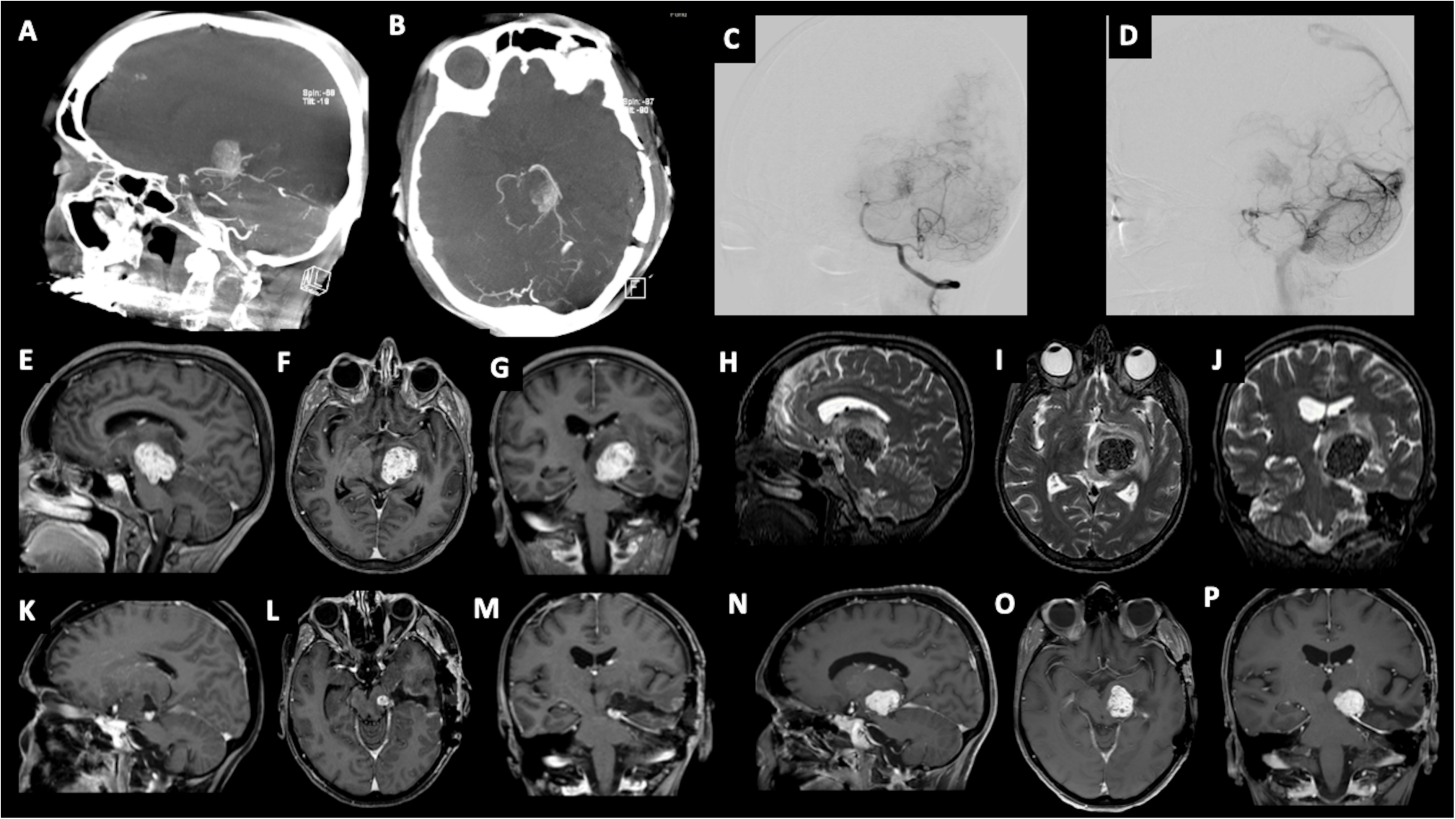
**(A–D)** Angiography imaging showing artero-venous blush lasting till late venous phase. The blush is fed by collaterals originating from P2 segment of the posterior cerebral artery (PCA), and left superior cerebellar artery (SCA). Preoperative post-contrast T1-weighted images in sagittal **(E)**, axial **(F)**, and coronal **(G)** views, showing the involvement of the tentorium hiatus and the extension to the left thalamo-mesencephalic region; Preoperative T2-weighted images in sagittal **(H)**, axial **(I)**, and coronal **(J)** views, showing the hypointensity of the tumor and the presence of perilesional edema. Post-operative post-contrast T1-weighted imaging showing residual tumor in sagittal **(K)**, axial **(L)**, and coronal views **(M)**; **(N-P)** 5-month follow-up post-contrast T1-weighted imaging showing disease recurrence.

**Figure 2:**
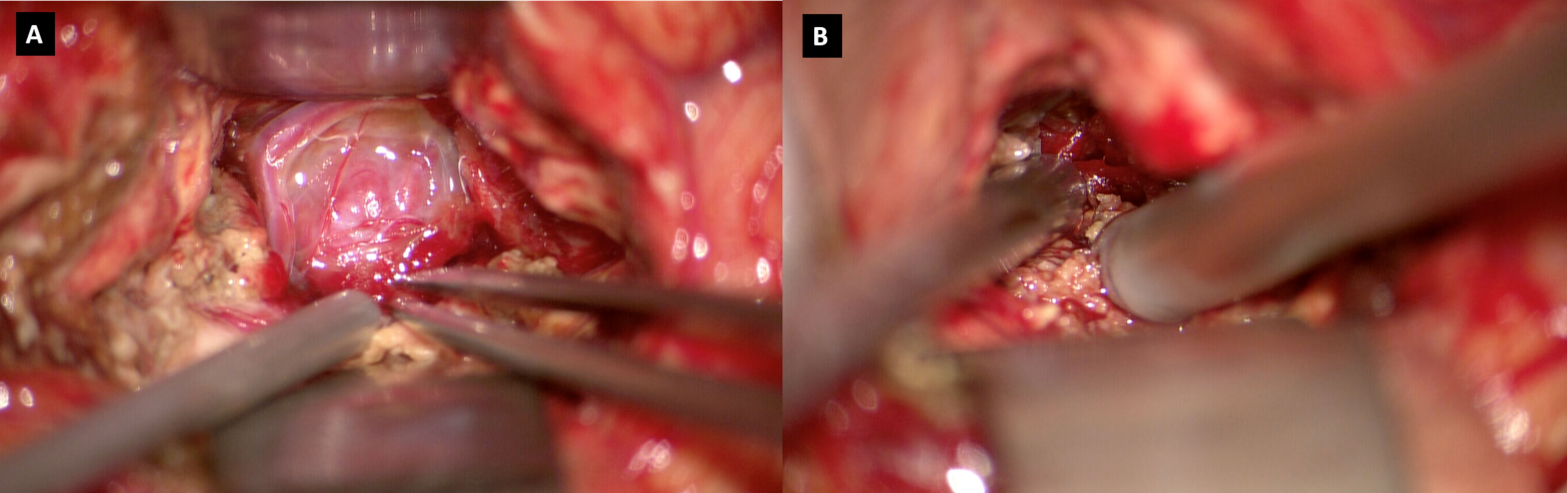
**(A)** Intraoperative visualization of the tumor, presenting as a hypervascularized mass in the tentorium hiatus; **(B)** Intraoperative use of *in vivo* confocal microscopy.

**Figure 3:**
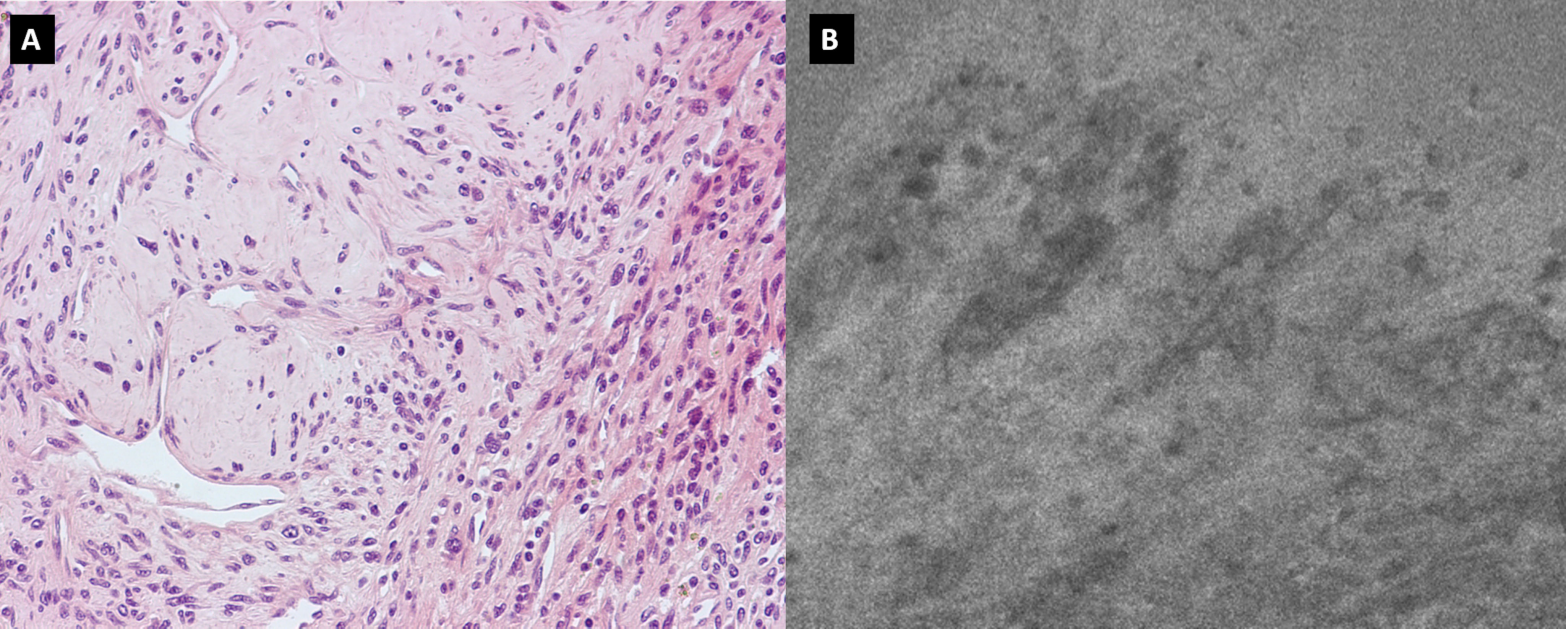
**(A)** H&H showing the presence of blood vessels, smooth muscle cells, and collagen tissue; **(B)** Confocal microscopy image showing neoplastic tissue characterized by significant neovascularization.

We present the case of a 60-year-old, right-handed, woman who suffered from a 9-months history of fatigue and weakness in the right limbs. Neurological examination revealed an ataxic gait, moderate right upper extremity dysmetria, and slight right hemiparesis. No previous history of CNS surgery or trauma was present, and no major comorbidities were reported. The patient underwent a preoperative brain MRI (**Figures 1E-J**) which disclosed a mass contiguous to the left tentorial free edge and extending into the ipsilateral thalamic and mesencephalic regions. The tumor was hypointense on T1-weighted images (WI) and on T2-WI (**Figures 1H–J**), and hyper/isointense on Fluid Attenuated Inversion Recovery (FLAIR) sequences. Postcontrast T1-WI showed heterogeneous enhancement of the lesion (**Figures 1E–G**), which was 31 millimeters in maximum diameter, and associated with moderate edema in the surrounding brain parenchyma. Magnetic Resonance spectroscopy (MRS) disclosed a reduction in N-acetyl-aspartate (NAA), and an increase in choline (Cho) and creatine (Cr) peaks with a reduction of Cho/NAA ratio in the tumor area, suggesting the glial nature of the lesion. In the suspicion of brain metastasis, a total body 18-Fluorodeoxyglucose Positron Emission Tomography-Computed Tomography (^18^F-FDG PET-CT) scan was acquired, displaying an increased tracer metabolism in the lesion without evidencing any extracranial pathologic uptake. Moreover, a brain computed tomography (CT) scan showed granular calcifications inside the lesion. Taking into consideration the neuroradiological findings, surgery was thought to be appropriate to both reduce the mass effect and make a definitive histological diagnosis. In the operating room, the patient was positioned supine and, under microscopic guidance, we decided to approach the lesion through a transtemporal tranventricular route in order to gain a wider control on both the lesion and its vascularization. The lesion was encountered in the left tentorial hiatus and appeared as an extraparenchymal, red and capsular mass with an arterialized surface (**Figure 2A**). The mass displayed a dense consistency and extended into the mesencephalic-thalamic region, occupying both the crural and ambient cisterna. At the beginning of the resection procedure, an excessive bleeding from the vascularised surface occurred, thus leading to an immediate interruption of the procedure. Postoperatively a brain Digital Subtraction Angiography (DSA) was performed (**Figures 1A–D**) and the presence of a thrombosed giant aneurysm was excluded. Moreover, DSA showed a delayed and intense arterio-venous blush fed by the P2 tract of posterior cerebral artery (PCA) and by superior cerebellar artery (SCA) afferents. Given that embolization of the tumor was considered unsafe by our interventional neuroradiology team, we decided to proceed with a re-operation through the same previous surgical route. During the second surgical procedure, we achieved a subtotal removal of the tumor without any surgical complication. Although the tumor exhibited an intense and homogenous fluorescence, the use of the dedicated filter (YELLOW 560) was not necessary because the pathologic tissue was already recognizable for its high vascularization. On the contrary, *In vivo* intraoperative confocal microscopy was useful in confirming the presence of the pathologic tissue, showing the presence of high cellularity and vascularized lesion (**Figure 2B**). The tumor specimen underwent histological examination, which reported the presence of blood vessels, smooth muscle cells, and collagen tissue (**Figure 3A**). Immunochemistry disclosed positivity for actin protein on smooth muscle cells, for CD31 and CD34 on endothelial cells, and for vimentin on mesenchymal tissue. On the contrary, STAT6, GFAP, and EMA antigens were not detected. The Ki67 index ranged from 4 to 5% and no necrosis was identified. These characteristics were ultimately consistent with the diagnosis of leiomyoma. Postoperatively, the patient displayed mild expressive dysphasia and moderate right hemiparesis. (**Figures 1K–M**). The patient was then transferred to a rehabilitation setting on the fifth day after surgery and both speech disturbance and strength deficit gradually ameliorated. Considering the subtotal resection and the benign histopathological features, a 5-months brain MRI was programmed. When MRI was performed, a significant disease recurrence was disclosed (**Figures 1N–P**). At this regard, we think that the disease recurrence was mainly due to the presence of residual tumor left after the second surgical procedure. Therefore, after a multidisciplinary discussion and taking into consideration the risk associated with reoperation, the patient was referred to the radiation therapy specialist at our Institution. The patient has completed a full cycle of radiation therapy, without any other neurological deficit, and is now waiting for the follow-up MRI.

## Material and methods

The case report has been described according to the CARE guidelines.

### Surgery protocol

The standardized surgical protocol of fluorescein-guided technique is based on i.v. SF injection at standard dose of 5mg/kg, by a central or peripheral venous line, immediately upon completion of the induction of general anesthesia (10). The surgery was performed with the aid of a surgical microscope equipped with an integrated fluorescent filter tailored to the excitation and emission wavelength of sodium fluorescein (YELLOW 560 – *Pentero 900; Carl Zeiss Meditec, Oberkochen, Germany*). During resection, the microscope could be switched alternatively from fluorescent to white-light illumination. Intra-operative fluorescein-assisted miniatured confocal laser endomicroscopy (CONVIVO^®^ system, Carl Zeiss, Meditec, Oberkochen, Germany) has been used.

### Literature review search strategy

A literature review search has been performed with the aid of the Preferred Reporting Items for Systematic Reviews and Meta‐analyses (PRISMA) statement guidelines, limited to the English language. SCOPUS, PubMed and Cochrane databases were queried using individual keywords and MeSH terms. A purposely defined search string was performed for PubMed, Scopus and Cochrane search: (“Leiomyoma”[Mesh]) AND “Central Nervous System”[Mesh], and for SCOPUS search: TITLE-ABS KEY (*intracranial* AND *angioleiomyoma*) AND (LIMIT TO (LANGUAGE, *“English”*)). The results were then limited to human subjects. After duplicate removal, title and abstracts were firstly screened and, for the papers deemed appropriate, full text was obtained and reviewed for appropriateness and extraction of data. Article references list was also examined to identify any other relevant study. Only studies dealing with the presence of intracranial ALM were included. Data from the included studies were extracted, organized, and analyzed. The qualitative assessment of the level of evidence of the papers extracted has been evaluated according to Oxford CEBM (11).

## Results

The results of the scoping literature review are summarized in **Table 1**. The literature review was based on articles published between 1994 and 2020. Since Lach et al. first reported a case of ALM, in 1994, 57 cases of ALM have been described (8). Considering the reported cases, intracranial ALMs are more common in men, with a male/female ratio of 1.9:1 (37 males; 19 females). The average age was 45,6 years (range: from 10 to 62 years). The most frequent clinical presentation was headache, which was described in 40% of cases. 7 patients (14%) with ALM presented with seizures. In sellar or parasellar lesions (38%), diplopia (20%) and visual impairment (24%) represented other primary clinical manifestations. Other symptoms, according to the location of the tumor, were motor deficits (14%), hearing loss (8%), vertigo, and tinnitus (6%). 6 cases (12%) were asymptomatic. Concerning the neuroradiological findings, ALMs were described as hypointense on T1-WI and/or hyperintense in T2-WI in 34 patients (59%). The tumor appeared hysointense in T1WI in 11 cases (19%). Heterogeneous contrast enhancement on T1-WI was observed in 6 cases (10%). In one case the lesion was depicted as a “solid” mass (1%). Regarding disease location, the sites of occurrence in order of frequency were: cavernous sinus with invasion (30%), tentorium (14%), sellar region (10%), temporal lobe (12%), falx cerebri (10%), optic nerve dura mater (5%), parietal lobe (5%), frontal lobe (3%), lateral ventricle (2%), cerebellopontine angle (2%), and occipital region (1%). The mean period of follow-up was 21,4 months after treatment and recurrence was not reported in any case. The main treatment was gross total resection (GTR), which was performed in 43 cases (86%). GammaKnife and radiation therapy represented adjuvant therapies in one case (2%) of subtotal resection.

**Table 1:** Literature Review Table.

Reference	Year	Age (years)Sex	Clinical Presentation	Dimension (cm)	Location	Neuroradiological Findings	FU(Months)	R	T	OCEBM
Rubiu et al. (**Current study)**	2022	60 F	Right arm and leg impairment	3.1	Left tentorium	T1: hypointense T2: hypointense FLAIR: hyper/isointense DWI: no restriction Heterogeneous CE CT scan: granular MR spectroscopy: NAA, Cho and Cr peaks reduction Cerebral Angiography Total Body PET-CT scan negative for other malignancies	5	None	GTR	Level 4
Tauziède-Espariat	2022	59 M	Not reported	22.8 mm (mean)	Left parietal	T1: hysointense T2: hyperintense	86 months (median)	None	GTR	Level 4
		46 M			Right temporal			None	GTR	Level 4
		59 F			Optic nerve dura mater			None	GTR	Level 4
		56 F			Optic nerve dura mater			None	GTR	Level 4
		51 M			CS			None	GTR	Level 4
		52 M			Right occipital			None	GTR	Level 4
		46 F			Optic nerve dura mater			None	GTR	Level 4
Ding et al.	2020	35 M	Left leg claudication	6.3x7.4x5.4	Lateral Ventricle	T1: hypointense T2: hyperintense	Not known	None	GTR	Level 4
Chen et al.	2020	59 M	Seizure Headache	1.3x1.1x1.1	Right TP	T1: hypointense T2: hyperintense	6	None	GTR	Level 4
Zhang et al.	2020	15 M	Right nose obstruction Headache	3 x 2.5 x 2.5 cm^3^	Right frontal cranial base	T1: hypointense T2: hyperintense	Not known	None	GTR	Level 4
Altieri et al.	2019	37 M	Incidental finding	3.9	Left tentorium	T1: hypointense T2: hyperintense	Not known	Not known	GTR	Level 4
Selbi et al.	2018	60 F	Diplopia	Not known	Right Meckel CS	T1: hypointense T2: hyperintense	48	None	STR GK	Level 4
Li et al.	2018	42 M	Vertigo Tinnitus Headache	3.1	Right CPA	CT: hyperdense	37	None	GTR	Level 4
		43 M	Incidental finding	2.9	Right tentorium	T1: hypointense T2: hyperintense	29	None	GTR	Level 4
		58 M	Incidental finding	2.6	Right PL	T1: hypointense T2: hyperintense	47	None	GTR	Level 4
		48 M	Diplopia	2.9	Right CS	T1: hypointense T2: hyperintense	46	None	GTR	Level 4
		41 M	Right CN VI palsy	2.9	Right CS	T1: hypointense T2: hyperintense	8	None	GTR	Level 4
		47 F	Left visual deficit	3.1	Left CS	T1: hypointense T2: hyperintense	33	None	GTR	Level 4
		58 M	Left visual deficit	1	Left Sellar Region	T1: hypointense T2: hyperintense	7	None	STR	Level 4
		53 M	Incidental finding	3	Falx Cerebri	T1: hypointense T2: hyperintense	5	None	GTR	Level 4
Liu et al.	2017	35 F	Right visual deterioration	Not known	Left CS Sellar Clival	T1: hypointense T2: hyperintense	12	None	GTR	Level 4
		48 F	Weakness of lower extremities	Not known	Tentorium	T1: hypointense T2: hyperintense	12	None	GTR	Level 4
		51 F	Visual deterioration Diplopia	Not known	Sellar Clival	T1: hypointense T2: hyperintense	12	None	GTR	Level 4
		19 M	Incidental finding	Not known	Not known	T1: hypointense T2: hyperintense	12	None	GTR	Level 4
Xiaofeng et al.	2016	36 M	Headache Diplopia	5x6x6	Right CS	T1: hypointense T2: hyperintense	3	None	STR RT	Level 4
Delgado et al.	2016	43 M	Hearing loss	1.4	Subtentorium	T1: hypointense T2: hyperintense	24	None	GTR	Level 4
Calle et al.	2016	43 M	Syncope	1.6	Falx Cerebri	T1: hysointense T2: hyperintense	Not known	Not known	GTR	Level 4
He et al.	2014	46 F	Headache Right Blepharoptosis	2	CS	Progressive CE	60	None	GTR	Level 4
		57 M	Headache Right Blepharoptosis Vision loss Diplopia	3	CS	Progressive CE	45	None	GTR	Level 4
		48 F	Headache Right severe ptosis	3	CS	Progressive CE	35	None	GTR	Level 4
		35 F	Headache Diplopia	2	CS	Progressive CE	2	None	GTR	Level 4
Lescher et al.	2014	40 M	Seizure Headache	Not known	Falx Cerebri	T1: hysointense T2: hyperintense	Not known	Not known	GTR	Level 4
Sun et al.	2014	51 F	Visual deficit	3x2.5x2.5	Sellar region	T1: hypointense T2: hyperintense	Deceased	–	GTR	Level 4
		49 M	Weakness of lower limbs	4.2x4.6x5.7	Subtentorium	T1: hypointense T2: hyperintense	12	None	GTR	Level 4
		77 M	Headache	1.6 x3.1x3.9	Left TL	T1: hypointense T2: hyperintense	12	None	GTR	Level 4
Teranishi et al.	2014	52 F	Right eye visual deficit	2.3	CS	T1: hypointense T2: hyperintense	Not known	Not known	GTR	Level 4
Li et al.	2014	23 F	Primary amenorrhea Visual deficit	5.5x5.5x7.7	CS and Sellar region	T1: hypointense	3	None	STR	Level 4
		62 M	Hypophasia Hypomnesis Altered consciousness	2.5x3.5x3.5	TL	T1: hysointense	Not known	Not known	GTR	Level 4
Zhou et al.	2013	62 M	Seizure	3.7x3.5x3.5	Middle fossa	T1: hypointense T2: hyperintense	7	None	GTR	Level 4
Gou et al.	2013	49 M	Weakness of both lower limbs	4.2x4.6x5.7	Tentorium	T1: hypointense T2: hyperintense	12	Not known	STR	Level 4
Conner et al.	2012	42 M	Headache	1	Right CH	T2: hyperintense	23	None	GTR	Level 4
		36 M	Headache	2.5	Falx Cerebri	CT: heterogeneous CE	26	None	STR	Level 4
Shinde et al.	2012	60 M	Headache Seizure	2	Right putamen Left hippocampus Optic nerve	T1: hypointense T2: hyperintense	Deceased	–	AUT	Level 4
Shi et al.	2012	60 M	Incidental finding	3.4x4.1	Right TL	T1: hypointense T2: hyperintense	6	None	GTR	Level 4
		36 F	Blurred vision Diplopia	2.8x2.5x1.2	Right sellar area	T1: hypointense T2: hyperintense	24	None	GTR	Level 4
Zu et al.	2012	60 M	Seizure	3.7x3.5x3	TL	T1: hypointense T2: hyperintense	Not known	Not known	GTR	Level 4
Xu et al.	2010	53 M	Headache Visual deficit	1	Sellar region	Not known	Not known	Not known	GTR	Level 4
Pepper et al.	2010	13 F	Hearing loss Headache	0.7	IAM	Not known	6	None	GTR	Level 4
Chongxiao et al.	2009	50 M	Headache Seizure	4x3x3	Falx Cerebri	T1: hypointense T2: hyperintense	18	None	GTR	Level 4
Gasco et al.	2009	43 M	Headache Blurred vision Dizziness Abnormal gait	4.4x3.9x3.9	Left CH	T1: hypointense T2: hyperintense	Not known	Not known	GTR	Level 4
Vijayasaradhi et al.	2008	10 F	Headache	4x3	FL	Not known	Not known	None	GTR	Level 4
Colnat et al.	2008	50 M	Headache	Not known	Left CS	T1: hypointense T2: hyperintense	72	None	GTR	Level 4
Karagama et al.	2005	47 F	Hearing loss	1	IAM	Solid	12	None	GTR	Level 4
Figueiredo et al.	2005	52 M	Headache Diplopia Visual deficit Facial Numbness	6	Right CS	T1: hysointense T2: hyperintense	Not known	Not known	GTR	Level 4
Kohan et al.	1997	Not known	Hearing loss Tinnitus	Not known	IAM	Not known	20	Not known	GTR	Level 4
Ravikumar et al.	1996	12 F	Headache Diplopia Seizure Left hemidystonia	Not known	Right head of CN	Not known	48	None	GTR	Level 4
Lach et al.	1994	47 M	Abnormal gait	2.7x2x2	Right PL	T1: hypointense T2: hyperintense	6	None	GTR	Level 4

CE, contrast enhancement; Cho, choline; CPA, cerebello pontine angle; CH, cerebral hemisphere Cr, creatine; CS, cavernous sinus; DWI, diffuse weighted imaging; GTR, gross total resection; FU, follow-up; IAM, internal acoustic meatus; MR, magnetic resonance; NAA, N-acetyl aspartate; PET, positron emission tomography; PL, parietal lobe; R, recurrence; T, treatment; TL, temporal lobe; TP, temporal pole. CEBM, Oxford Center for Evidence Base Medicine.

## Discussion

CNS ALM represents a rare disease, and no common agreement exists on its diagnostic and surgical management. We provide a case report with a short-term recurrence and a thorough pre-operative and intra-operative illustration, with the aid of confocal microscopy. A scoping literature review is also presented to summarize and augment the level of evidence for the management of CNS ALM. ALM is a grayish-brown soft tissue tumor composed of vascular channels and stroma, in which loose smooth muscle bundles and collagen are housed (8, 12). Microscopically, thick-walled vessels are surrounded by fascicles of eosinophilic spindle cells (12). These histological features are confirmed by immunostaining through positivity to alpha-actin and h-caldesmin, which represent specific markers for smooth muscle cells. Histologic features and immunostaining may facilitate differential diagnosis between ALM and meningiomas, arteriovenous malformations, and solitary fibrous tumors. Although Hachisuga et al. (6) found mature fat cells within a specimen of intracranial ALM, the present case was characterized by the presence of blood vessels, smooth muscle cells, and collagen tissue, without any evidence of fat tissue. CNS ALM usually increases in size over a period of months to years before causing any clinical manifestation. Even when present, clinical manifestations are nonspecific and mostly related to the space-occupying mass. In our case, the tumor was responsible for a slight right hemiparesis due to its proximity to the left cerebral peduncle. Because of their uncommon presentation and atypical neuroradiologic features on CT and MRI, intracranial ALMs are often misdiagnosed (9). Differential neuroradiological diagnosis includes meningiomas, schwannomas, cavernous hemangioma, solitary fibrous tumors, and dural metastasis (9). ALM usually appears as a hyperintense or isointense lesion on T1WI and shows hyperintensity on T2WI. Postgadolinium enhancement is also featured. In our case, the tumor appeared hypointense on T1WI and T2WI and hyper/isointense on FLAIR images. Post-gadolinium scan showed an intense and heterogeneous contrast enhancement with moderate perilesional brain edema (**Figure 1**). The maximal tumor diameter was 31 millimeters. MRS highlighted a low NAA/Cho ratio in the pathological area, mistakenly suggesting a glial nature of the lesion. Similarly to other intracranial tumors such as meningiomas and gliomas, in the case of ALM surgical resection represents the cornerstone of therapy. In our case, we decided to approach the lesion through a transtemporal tranventricular route in order to gain a wider control on the lesion. Although a sub-temporal intradural approach could have been performed, we decided to not choose it because of its associated need to retract the dominant temporal lobe. Moreover, the transtemporal tranventricular transchoroidal approach gave us the opportunity to violate only part of the inferior temporal gyrus. Finally, the lateral supracerebellar transtentorial approach was not performed because it could not allow a complete control of the vascular structures. The lesion was then identified in the left tentorial hiatus, appearing as an extraparenchymal, red and capsular mass with an arterialized surface (**Figure 2A**). The mass displayed a dense consistency and extended into the mesencephalic-thalamic region, occupying the crural and ambient cisterna. At the beginning of the surgical resection, an excessive bleeding from the vascularised lesion occurred, leading us to abort the procedure. At this regard, surgical resection may be challenging even to the most experienced surgeon, as reported by Gasco et al. (13, 14), because of the highly vascularization of intracranial ALMs. The bleeding propensity of ALM raises questions about the usefulness of preoperative embolization. At this regard, we suppose that preoperative embolization of the tumor may be considered in cases of complex vascular architecture lesions and proximity to large vessels (i.e., cavernous sinus), in which the embolization procedure may avert the burden of intraoperative bleeding. Nonetheless, despite the application of this procedure, significant bleeding from the tumor still represents a frequent complication (15, 16). After aborting the first surgical attempt, we performed a DSA that excluded the presence of any thrombosed aneurysm and provided us the needed information about vascular afferents to the tumor. The second surgical procedure was conducted through the same previous surgical route, achieving a subtotal removal of the tumor without any surgical complication. During the resection, the lesion displayed an intense and homogenous fluorescence, and the use of the dedicated filter (Yellow 560) was not necessary because the pathologic tissue was already recognizable for its high vascularization. Confocal laser endomicroscopy (CLE) was implemented and showed both abnormal vessels and neoplastic proliferation. At this regard, we would like to highlight the usefulness of the CLE in differentiating the neoplastic portion of the tumor from its vascular component. We also underline that CLE could have represented a useful intraoperative adjunct to exclude a vascular malformation in the first setting, thus preventing us from aborting the surgery. In patients with ALM, post-surgical complications such as hydrocephalus, seizure, and visual impairment (17–21) have been reported. Our patient developed mild expressive dysphasia and moderate right hemiparesis. Postoperative brain MRI showed a residual tumor located in the free tentorial edge and firmly attached to the left midbrain. Despite the presence of residual disease, considering its histological benignity and after a multidisciplinary neurooncological board, we decided for a follow-up with a brain MRI, which showed a significant recurrence at 5 months after surgery (**Figures 1N–P**). The patient was then referred to our radiation therapy specialist. The recurrence of the disease has not been described in the literature so far. As described by Xiaofeng et al., the postoperative residual disease can be treated by Cyber-knife (12). Such treatment should also be considered in cases at high risk for bleeding or when large vascular structures are involved. On the ground of our case, we may suggest a closer neuroradiological follow-up and, in selected cases, adjuvant radiation therapy in residual disease to prevent significant tumor recurrence. Because of the rarity of the lesion, a larger sample with multicentric collaborative studies is needed to reach more significant conclusions on the best adjuvant treatments. Moreover, given the fact that ALM usually does not exhibit any aggressive biological behavior, the identification of prognostic factors suggestive of disease recurrence is also needed. Immunotherapy has been proposed as a therapeutic option by Shinde et al., in their peculiar multifocal ALM report (20) but further clinical data are needed to confirm its clinical usefulness. On the other hand, Li et al. (20) opted for biopsy and radiosurgery in the case of an ALM located in the sellar region.

## Conclusion

Intracranial ALM represents a rare and understudied CNS tumor. We report the first case of CNS ALM undergoing an intraoperative confocal endomicroscopy with the potential usefulness to discriminate an unexpected vascular malformation from a highly vascularized neoplastic lesion. We provide a comprehensive radiological and histopathological evaluation along with a literature review. Moreover, this case could suggest the need to consider radiation therapy as an adjuvant modality treatment in ALM subtotal removal.

## Data availability statement

The raw data supporting the conclusions of this article will be made available by the authors, without undue reservation.

## Ethics statement

Ethical review and approval was not required for the study on human participants in accordance with the local legislation and institutional requirements. The patients/participants provided their written informed consent to participate in this study. Written informed consent was obtained from the individual(s) for the publication of any potentially identifiable images or data included in this article.

## Author contributions

EC, FA and PF performed the clinical assessment. ER, EC and GB critically reviewed the literature and drafted the manuscript. All authors were responsible for important intellectual content. All authors contributed to the article and approved the submitted version.
