# Supplementary figures and images for: Diagnostic and surgical management of primary central nervous system angioleiomyoma: A case report and literature review
Source: Front Oncol. 2022 Dec 16;12:1072270. doi: 10.3389/fonc.2022.1072270 (PMC9800865; doi:10.3389/fonc.2022.1072270)

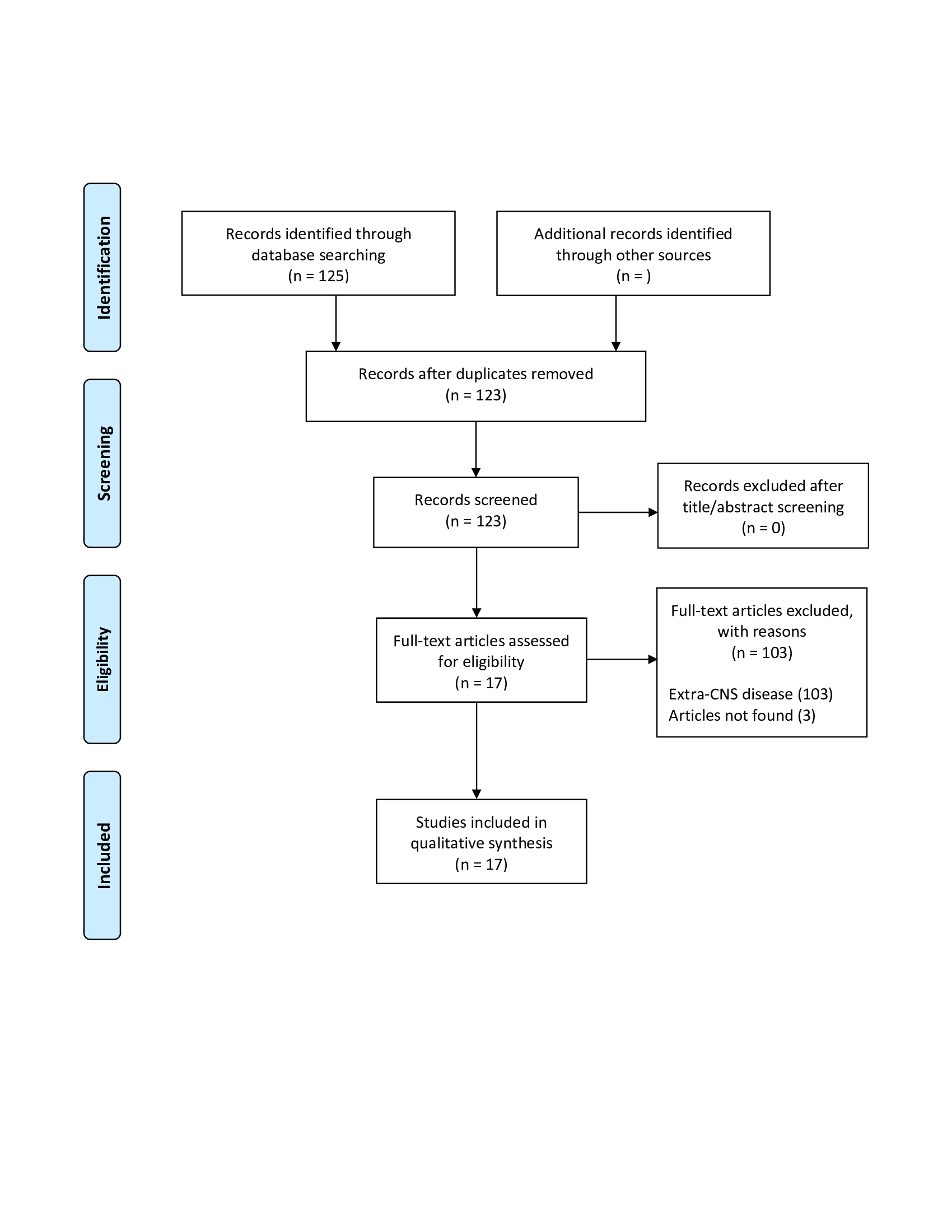

Supplement: Supplementary file 1 [file Image_1.tiff]

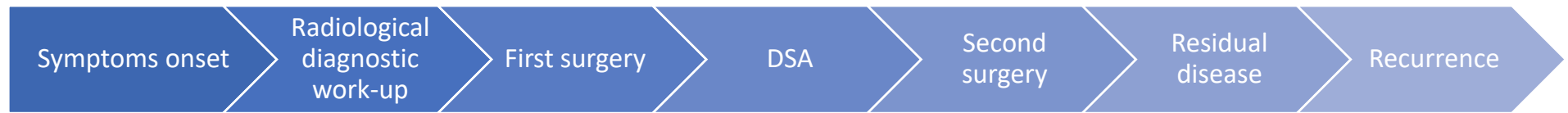

July 2021

April 2022

May 2022

September 2022

Supplement: Supplementary file 2 [file Image_2.pdf]
